# High-Resolution Melting Analysis for Rapid Detection of Sequence Type 131 Escherichia coli

**DOI:** 10.1128/AAC.00265-17

**Published:** 2017-05-24

**Authors:** Lucas B. Harrison, Nancy D. Hanson

**Affiliations:** Department of Medical Microbiology and Immunology, Creighton University, Omaha, Nebraska, USA

**Keywords:** HRM, MLST, multiplex, ST131, molecular epidemiology

## Abstract

Escherichia coli isolates belonging to the sequence type 131 (ST131) clonal complex have been associated with the global distribution of fluoroquinolone and β-lactam resistance. Whole-genome sequencing and multilocus sequence typing identify sequence type but are expensive when evaluating large numbers of samples. This study was designed to develop a cost-effective screening tool using high-resolution melting (HRM) analysis to differentiate ST131 from non-ST131 E. coli in large sample populations in the absence of sequence analysis. The method was optimized using DNA from 12 E. coli isolates. Singleplex PCR was performed using 10 ng of DNA, Type-it HRM buffer, and multilocus sequence typing primers and was followed by multiplex PCR. The amplicon sizes ranged from 630 to 737 bp. Melt temperature peaks were determined by performing HRM analysis at 0.1°C resolution from 50 to 95°C on a Rotor-Gene Q 5-plex HRM system. Derivative melt curves were compared between sequence types and analyzed by principal component analysis. A blinded study of 191 E. coli isolates of ST131 and unknown sequence types validated this methodology. This methodology returned 99.2% specificity (124 true negatives and 1 false positive) and 100% sensitivity (66 true positives and 0 false negatives). This HRM methodology distinguishes ST131 from non-ST131 E. coli without sequence analysis. The analysis can be accomplished in about 3 h in any laboratory with an HRM-capable instrument and principal component analysis software. Therefore, this assay is a fast and cost-effective alternative to sequencing-based ST131 identification.

## INTRODUCTION

Increasing reports of antibiotic-resistant bacteria represent a global challenge to human health care (1). One strain that has emerged as an international multiresistant high-risk clone is sequence type 131 (ST131) Escherichia coli (2). This sequence type has been associated with fluoroquinolone resistance and the global dissemination of CTX-M antibiotic resistance genes and has recently been shown to harbor *mcr-1*-bearing plasmids (3, 4). Previous studies have shown that treatment with fluoroquinolones or cephalosporins will select for ST131 E. coli (5). Therefore, to increase our ability to detect ST131 E. coli and improve surveillance, rapid and cost-effective methods for the detection of this international multiresistant high-risk sequence type are crucial (6). Tracking the spread of this particular clonal complex has been made possible through ST identification by whole-genome sequencing (WGS) and multilocus sequence typing (MLST) (7–9). These methods, while effective, can be expensive, labor intensive, and time consuming. One technology that has recently been adapted to evaluate sequence differences between DNA samples uses high-resolution melting (HRM) analysis (10).

HRM is a technique used to determine whether two PCR amplicons of similar size have identical sequences. This technique is similar to quantitative PCR (qPCR) in that it amplifies a gene target in the presence of a fluorescent reporter dye. Following amplification, the product is exposed to an increasing temperature gradient to denature and reduce the helicity of the double-stranded oligonucleotide, releasing the fluorescent dye. Once released, the dye undergoes a conformational change that reduces the amount of fluorescence produced. An HRM-capable thermocycler will record the fluctuations in fluorescence and produce a melt curve unique to the sequence of the amplicon analyzed. This technique is capable of comparing the similarity of two amplicons because the melt curve of each oligonucleotide is determined by its nucleotide sequence, oligonucleotide length, and primary structure (11, 12). The weak A-T bond is disrupted at a lower temperature than the G-C bond, and therefore, A-T–rich regions of the amplicon denature at lower temperatures than G-C–rich regions (13). The distribution of these A-T– or G-C–rich regions of the oligonucleotide dictates the resulting melt curve and can be used to compare the similarity of amplicons from multiple samples.

HRM has previously been employed to compare the genetic similarities in strains of Enterococcus faecium, Staphylococcus aureus, Klebsiella pneumoniae, Acinetobacter baumannii, Pseudomonas aeruginosa, and Enterobacter spp. (14). These methods, however, require lengthy preparation and generate a large number of oligonucleotides that can obscure differences between the genomes (15). One alternative is a targeted melting analysis of MLST amplicons. By selecting specific genes for amplification, a targeted HRM-based methodology reduces the sample preparation requirements such that they are similar to those of a multiplexed qPCR. Furthermore, with the development of selective dyes, such as LCGreen, SYTO-9, and EvaGreen, HRM analysis can discriminate between the larger amplicons required for MLST, ranging from ∼600 to 900 bp (16–20). The purpose of this study was to develop and validate the single-nucleotide polymorphism (SNP)-level discriminatory power of HRM to enable rapid and cost-effective differentiation of ST131 E. coli from non-ST131 E. coli.

## RESULTS

### Development of the methodology.

**FIG 1:**
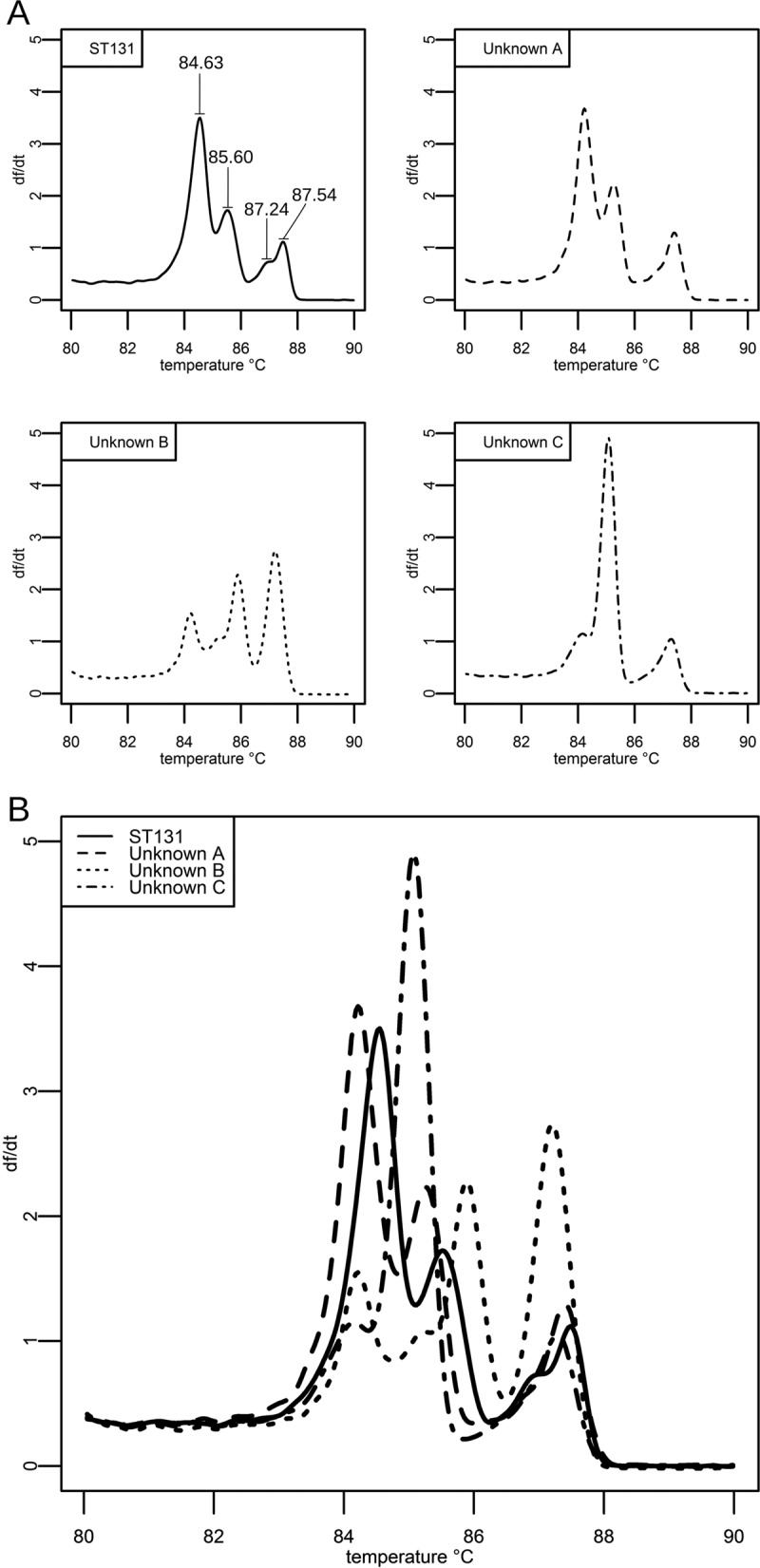
Melt curve comparisons between known and unknown sequence types. (A) HRM profiles of 4 clinical isolates (solid line, 1 ST131 isolate; dashed lines, 3 isolates of unknown sequence type) from the *adk*, *gyrB*, *mdh*, and *recA* multiplexed reaction mixture. (B) The four individual melt profiles from panel A are presented on the same graph. By running a known ST131 sample as a control, non-ST131 isolates can be visually identified by their lack of similarity to the ST131 melt profile.

The derivative melt curves of singleplex MLST amplicons matched the melt curves predicted by the uMelt software. Next, the derivative melt curves from multiplexed HRM reactions were compared between ST131 and non-ST131 E. coli (see Fig. S1 in the supplemental material). A direct comparison of the derivative melt curves between strains showed that all ST131 isolates shared similar profiles that were distinct from the profiles of non-ST131 isolates. With this multiplexed reaction, ST131 identification requires the peaks to be defined by temperature, as well as by the temperature difference between the peaks. The temperatures of the comparator ST131 melt profile peaks were evaluated across all validation runs. The profile peak temperatures for ST131 controls in the *adk*, *gyrB*, *mdh*, and *recA* master mixture were 84.63 ± 0.16°C, 85.60 ± 0.13°C, 87.24 ± 0.33°C, and 87.54 ± 0.09°C (Fig. 1). For the *fumC*, *icd*, and *purA* master mixture, the ST131 control peak temperatures were 85.30 ± 0.30°C, 86.40 ± 0.25°C, and 87.75 ± 0.15°C. To account for differences in profile peak temperatures between runs, we also investigated the temperature difference between peaks within the multiplexed reactions. The temperature differences between the peaks in the *adk*, *gyrB*, *mdh*, and *recA* master mixture were 0.99 ± 0.04°C between peaks 1 and 2 and 1.69 ± 0.29°C between peaks 2 and 3, while peaks 3 and 4 showed a difference of 0.49 ± 0.05°C. In the *fumC*, *icd*, and *purA* master mixture, the temperature difference was 1.12 ± 0.65°C between peaks 1 and 2, while peaks 2 and 3 showed a difference of 1.36 ± 0.11°C.

**FIG 2:**
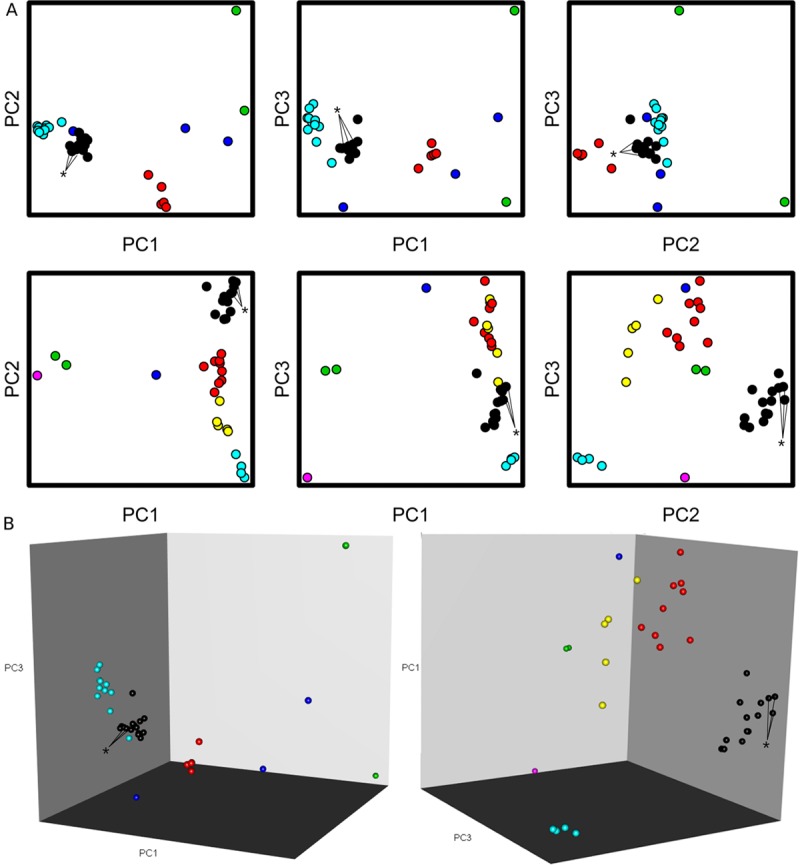
Principal component analysis of E. coli isolates of unknown sequence type. (A) PCA visualization of the E. coli MLST amplicon residual melt profile data from the ABI 7500 Fast PCR thermocycler using the open source R programming language. Data points represent a single run of one 96-well plate from the ABI 7500 Fast thermocycler containing both master reaction mixtures of 45 clinical isolates and 3 comparator ST131 E. coli isolates. Comparator E. coli isolates are indicated by asterisks. Isolates were grouped into clusters based on similarity of PCA profiles, and clusters were subsequently color coded. Black clusters are identified as ST131 isolates, while other colors indicate groups of unknown sequence types. (B) The three 2-D PCA plots for each master reaction mixture were combined into an interactive 3-D model to facilitate analysis. As in panel A, the black clusters represent ST131 E. coli isolates.

Multiplexed melt curves of ST131 E. coli identified 66/66 ST131 isolates on both the Rotor-Gene Q and ABI 7500 system, displaying 100% sensitivity. One false positive on the Rotor-Gene Q and two false positives on the ABI 7500 were recorded. Principal component analysis (PCA) of HRM data using ScreenClust HRM software (Fig. 2) identified 66/66 ST131 E. coli isolates but also incorrectly identified three non-ST131 isolates from the 191 isolates evaluated as ST131. A comparison of the data (Table 1) showed that all three analyses performed with 100% sensitivity and that the derivative melt curve analysis had greater specificity than the ScreenClust HRM software analysis.

**TABLE 1:** Cross-platform sensitivities and specificities^*a*^

Platform	No. of isolates with indicated result determined by derivative melt curve^*b*^ (no. determined by PCA^*c*^)				Sensitivity (%) of:		Specificity (%) of:	
	True positive	False positive	True negative	False negative	HRM	PCA	HRM	PCA
Rotor-Gene Q	66 (66)	1 (3)	124 (122)	0 (0)	100	100	99.2	96.8
ABI 7500 Fast	66	2	123	0	100		98.4	

a Blind validation results of the HRM methodology evaluation of the panel of 191 clinical isolates, consisting of 66 ST131 and 125 non-ST131 E. coli isolates.

b Determined by comparing the derivative melt curve to an ST131 standard.

c Determined by ScreenClust HRM software principal component analysis and clustering.

## DISCUSSION

Antibiotic challenge from fluoroquinolones or cephalosporins used in empirical therapy selects for the survival of ST131 E. coli (5). Rapid diagnostic tools for detecting this strain are needed to prevent the spread of this prolific strain and inform strategies to impact the clinical outcomes of patients. Several in-house E. coli sequence typing methods have recently been developed to address this need. While these methods include running a PCR product on a gel, probe-based qPCR, and HRM, they depart from the Achtman MLST schema for E. coli (21–25). Conventional MLST methods provide high sensitivity and specificity but require the seven PCR products to be evaluated by gel electrophoresis to confirm amplicon generation, followed by sequence analysis for each of the seven amplicons individually (26). Alternative approaches based on sequencing incur costs in time or money that make screening for ST131 E. coli in a large sample size an expensive endeavor. Following DNA extraction and sample dilution, the roughly 2-h HRM analysis outlined in this study reduces reagent costs to ∼$5.00/sample, in comparison to ∼$80.00/sample (Table 2) using conventional PCR amplification and sequencing to identify ST131 E. coli. As a consequence of the differences in speed and price, this gel-free HRM method facilitates screening for ST131 E. coli in large sample populations.

**TABLE 2:** Comparison of methodologies

Procedure	Outline	Cost for analysis^*a*^	Time for results	Result
MLST	PCR, column purification, sample dilution, offsite sequencing	$78.96^*b*^	2 to 3 days	Sequence type classification of all isolates
HRM screen	Sample dilution, HRM reaction	$4.28 to $5.18^*c*^	∼3 h	Identification of ST131 E. coli

a Cost for analysis at time of article submission.

b Price cited includes the cost of PCR reagents, column purification, and Sanger sequencing for each of the 7 MLST genes.

c Price cited is the cost of the Qiagen DNeasy blood and tissue kit and HRM master mix from Qiagen or Thermo Fisher Scientific for both master mixes.

This methodology was initially validated on a Qiagen Rotor-Gene Q platform using the manufacturer's HRM Type-it kit. To ensure the method would work on other platforms, an additional validation was performed on an Applied Biosystems 7500 Fast system with the manufacturer-recommended MeltDoctor HRM mixture. The commercially available ScreenClust HRM software is incompatible with the HRM output from the Applied Biosystems 7500 Fast system, and only the derivative melt curves were evaluated on this platform.

PCA is a data reduction tool that can condense complex data sets into simple visual representations (27, 28). In this study, visual analysis made it possible to evaluate the melt profiles of 36 samples at once without requiring individual comparisons to an ST131 melt curve standard. PCA of these samples maintained the same sensitivity as the derivative melt curve but had a lower specificity. The scatterplots produced by PCA reflect variance between the samples, and as such, analyzing a large number of closely related sequence types may prevent distinct clusters from being produced. In this case, PCA should be used to quickly rule out samples that are not ST131 isolates and be followed by derivative melt curve analysis of the remaining samples.

During our analysis, we observed that the mass of DNA in the reaction mixture affected the shape of the derivative melt curve. DNA quantification prior to performing the HRM procedure is a necessary step to avoid introducing error. The Qiagen DNeasy blood and tissue preparation kit with RNase treatment was sufficient to allow DNA quantification using UV spectrophotometry. Altering the amount of DNA used in the PCR amplification step beyond a 2-fold concentration affects both the observed melting temperature and the magnitude of the melt peaks. The technical error of using too much DNA can be identified by the derivative melt curve analysis because the resulting curve is similar to the ST131 standards. The difference in peak profiles caused by loading error, however, will affect the PCA values for the sample and cause it to be placed askew on the resulting cluster plot.

This real-time multiplex PCR followed by HRM analysis is a one-step PCR that can identify ST131 E. coli from other sequence types in roughly 3 h. If direct sequencing of the amplicons is required to determine the specific subsets of ST131 E. coli, this initial screen will differentiate the non-ST131 isolates and reduce the number of organisms that need to be further evaluated. Furthermore, data analysis software from either commercial vendors or open source providers is available to streamline the analysis on Windows, Mac, or Linux operating systems. This HRM methodology can be performed on multiple platforms with a high degree of sensitivity and specificity and is both faster and less expensive than conventional sequence typing methods.

## MATERIALS AND METHODS

### Culture conditions and DNA extraction.

Six ST131 E. coli strains obtained from the University of Pittsburgh Medical Center and 6 non-ST131 E. coli strains (1 each of ST167, ST182, ST410, ST439, ST648, and ST2261) were selected to develop this methodology (29). DNA was extracted from 10-ml overnight cultures grown in Mueller-Hinton broth (MHB) using a Qiagen DNeasy blood and tissue kit. The concentration and purity of the DNA were evaluated with a UV spectrophotometer (BioTek Eon), and the samples were diluted to 10 ng/μl.

### Singleplex PCR amplifications.

Singleplex endpoint PCRs of ST131 DNA template and individual MLST primer sets were performed using the Qiagen Type-it HRM PCR kit in a final volume of 25 μl with 10 ng of DNA template and 12.5 pMol each primer (30). DNA quantification prior to performing the HRM procedure is a necessary step to avoid introducing error due to modifications in the shape of the melt curves. The cycling conditions consisted of a 5-min denaturation at 95°C, followed by 30 cycles of a 10-s denaturation at 95°C and 30 s of annealing at 51°C and then by 10 s of extension at 72°C. Amplicons were analyzed by HRM at a 0.1°C resolution from 50 to 95°C to determine the absolute melt curve of each amplicon. The derivative melt curves of MLST amplicons were compared to the melt curves predicted by uMelt software, developed at the University of Utah, to confirm sequence identity. PCR amplicons were also separated on a 1% agarose gel and visualized with ethidium bromide to confirm that only a single product of the expected size was amplified.

### HRM analysis of multiplexed MLST amplicons.

A single sevenplex reaction mixture of all MLST primer sets was initially tested to differentiate ST131 from non-ST131 E. coli. This multiplex reaction mixture failed to have sufficient discriminatory power, and the reaction mixture was divided into a pair of three- and fourplex reaction mixtures consisting of the primers for amplification of *fumC*, *icd*, and *purA* (primer set 1) and those for *adk*, *gyrB*, *mdh*, and *recA* (primer set 2). The primer concentrations remained the same as in the singleplex reaction mixtures, and the primer combinations were selected to minimize melt curve overlap between individual amplicons. Following amplification of these primer sets against both ST131 and non-ST131 templates under the cycling conditions described above, the MLST amplicons were subjected to HRM analysis in the optimized range of 80 to 95°C. The derivative melt curves generated were compared between sequence types to evaluate the discriminatory power of this methodology. Both melt curves from E. coli isolates of unknown sequence type were compared to ST131 melt profiles (Fig. 1). The unknown sequence type was designated ST131 if the melt curves from both master mixtures matched the ST131 profile.

### Validation of the methodology.

A blinded study using 191 E. coli clinical isolates of unknown sequence types was performed to validate the methodology. Three ST131 positive controls were included with each analysis to differentiate ST131 E. coli from non-ST131 E. coli. To validate that the unknown-sequence-type samples identified as ST131 by HRM analysis were ST131, the sequences of their *gyrB* and *mdh* genes were determined by Sanger sequencing. These two genes provide sufficient discrimination to identify the ST131 clonal complex (21). Validation of the method by melt curve analysis was performed on both the Rotor-Gene Q 5-plex HRM (Qiagen) and the ABI 7500 Fast PCR Thermocycler (Applied Biosystems) using the manufacturer-recommended HRM-Type-it (Qiagen) and MeltDoctor HRM kit (Applied Biosystems), respectively. The sensitivity and specificity of this methodology were compared against those of the sequence typing standard of MLST amplicon sequencing.

The sequences of the MLST amplicons produced in this study were determined offsite at Functional Biosciences, Inc. (Madison, WI), by Sanger sequencing (BigDye version 3.1, ABI 3730xl). Samples were prepared to company submission standards, and the nucleotide calling at each position was determined by a Phred score greater than 20. The resulting sequences were then identified using the E. coli MLST database maintained at the University of Warwick (Coventry, UK).

### PCA of multiplexed MLST amplicons.

As an alternative to manually comparing the derivative melt curves of each sample, Qiagen ScreenClust HRM software was employed to differentiate between sequence types using clustering analysis. After selecting samples by master mixture, the software automatically processes the data by principal component analysis (PCA) and clusters the samples into groups based on the variance in fluorescence between clinical isolates. Briefly, a residual melt curve was automatically constructed from the mean values of normalized data for each isolate. The differentials of the residual melt curves were plotted and analyzed by PCA to construct a feature that reflected the greatest variance of each isolate from the group. The first three principal components in PCA reflect the greatest degree of variance, and these values were graphed onto a scatterplot. The principal components were further processed by the ScreenClust HRM software to group isolates with similar variances into clusters. With the exception of selecting the isolate samples for analysis, the ScreenClust HRM software automates the analysis. While ScreenClust HRM software analysis is currently only available for the Qiagen platform, open-source software may be employed to perform PCA and clustering analysis as described in the supplemental material.

The ABI 7500 system does not include PCA software, but HRM data from this platform can be exported and analyzed with third-party software. One free, open-source option is the R software environment, available from the R Project for Statistical Computing. PCA is part of the functionality of the R 3.2.2 base package, and the *rgl* and mclust packages were used for three-dimensional (3-D) visualization and clustering, respectively (31–34). Performing the analysis with these packages as described in the supplemental material allows the user to both identify ST clusters and represent them in 2-dimensional (2-D) scatterplots (Fig. 2A) and a representative 3-D interactive model (Fig. 2B).

## Supplementary Material

Supplemental material
