# Supplementary material for: High-Resolution Melting Analysis for Rapid Detection of Sequence Type 131 Escherichia coli
Source: Antimicrob Agents Chemother. 2017 May 24;61(6):e00265-17. doi: 10.1128/AAC.00265-17 (PMC5444143; doi:10.1128/AAC.00265-17)
Supplement: Supplemental material [file supp_61_6_e00265-17__index.html]

High-Resolution Melting Analysis for Rapid Detection of Sequence Type 131 Escherichia coli — Supplemental material 

# High-Resolution Melting Analysis for Rapid Detection of Sequence Type 131 Escherichia coli

## Supplemental material

- Supplemental file 1 -

  Supplemental material

  PDF, 11M
