# Supplementary material for: High-Resolution Melting Analysis for Rapid Detection of Sequence Type 131 Escherichia coli
Source: Antimicrob Agents Chemother. 2017 May 24;61(6):e00265-17. doi: 10.1128/AAC.00265-17 (PMC5444143; doi:10.1128/AAC.00265-17)
Supplement: Supplemental material [file AAC.00265-17_zac007176265s1.pdf]

Principal Component Analysis of HRM Data Using R

Construction of the Differential Melt Curve

The differential melt curve is automatically calculated in both the Rotor-Gene Q software suite and the ABI HRM suite. If an alternate platform is used that does not generate a differential melt curve, the temperature and fluorescence data can be analyzed in the R base package. The data were organized into a table with the column headings of temperature and the individual sample IDs. The identity of each curve was then displayed with the ‘identify’ command.

```
temperature <- exported.data.file$temperature
sample.fluorescence <- exported.data.file$sampleID
differential.curve.y <- diff(sample.fluorescence)/diff(temperature)
x.range <- seq(temperature[1], tail(temperature, n = 1), length.out
=length(differential.curve.y))
plot(x.range, (-(differential.curve.y)), xlab = ‘temperature’, ylab =
‘df/dt’, type = ‘l’)
df.exported.data.file.notemp <- as.data.frame(exported.data.file)
df.exported.data.file.notemp$temperature <- NULL
identify(x.range, (-(differential.curve.y)), labels
=colnames(df.exported.data.file.notemp)
```

Conctruction of Residual Melt Curve

Data for constructing the residual melt curve were organized as follows: individual isolates served as the header for each column and temperature readings labeled each row. The

fluorescence matrix was then populated with the corresponding fluorescent reading for each x-axis isolate and y-axis temperature. Each column was individually normalized to a range of [0:1] by first subtracting the lowest value from the each column and then dividing by the highest value. Once the data were normalized, the mean fluorescence across all isolates for each temperature point was calculated and used to create the mean melt curve. The values for the mean melt curve were subtracted from the values of each isolate to produce the residual melt curve specific to each isolate.

PCA and Clustering

The residual melt curves from each master mix was analyzed by PCA with a covariance matrix instead of a correlation matrix. Analysis as a correlation matrix incorporates a normalization step and is used when the values analyzed are orders of magnitude apart. As the HRM data had been normalized in a previous step, the covariance matrix was chosen over the correlation matrix.

```
PCA.analysis.file <- prcomp(fluorescence.matrix.MasterMix1[,1:36], cor = FALSE)
```

The first three principal components were then grouped into clusters using the agglomerative clustering algorithm within the R package, ‘mclust’.

```
cluster.data <- Mclust(PCA.analysis.file$rotations[,1:3])
df.PCA.MM1 <- as.data.frame(PCA.analysis.file$rotations[,1:3])
```

Principal component data is stored in the 'rotations' list of the PCA file generated in the previous step, and the addition of [1:3] instructs the program to select only the first three principal components for cluster analysis. Cluster designation of the isolates is stored in the 'classification' list.

Visualization

Images of the three 2D scatterplots of the principal components were created using the R ‘plot’ command and color coded according to their clustering designation. Subsequent identification of each sample from the scatterplot was made possible through the use of the ‘identify’ command.

```
plot(df.PCA.MM1[,1:2], bg=cluster.data$classification, col=1, pch=21,
box(lwd=4), cex.lab=2.0, xaxt='PC1', yaxt='PC2', cex=1)
identify(PCA.MM1[,1:2], labels = rownames(PCA.MM1))
plot(df.PCA.MM1[,2:3], bg=cluster.data$classification, col=1, pch=21,
box(lwd=4), cex.lab=2.0, xaxt='PC1', yaxt='PC2', cex=1)
identify(PCA.MM1[,2:3], labels = rownames(PCA.MM1))
plot(df.PCA.MM1[,1], bg=cluster.data$classification, col=1, pch=21,
box(lwd=4), cex.lab=2.0, xaxt='PC1', yaxt='PC2', cex=1)
identify(PCA.MM1[,1], PCA.MM1[,2], labels = rownames(PCA.MM1))
```

Alternatively, a black and white 2D PCA scatterplot may be created by substituting the `cluster.data$classification` into the `pch` argument (S.Fig 2)

```
plot(df.PCA.MM1[,1:2], col=1, pch=(cluster.data$classification + 19),
cex.lab=2.0, xlab='PC1', ylab='PC2', cex=1)
```

Finally, a 3D model of these three 2D scatterplots was created with the ‘plot3d’ command, and the points identified using the ‘identify3d’ command.

```
plot3d(PCA.MM1, col=fit2$classification, size=.75, type='s')
identify3d(PCA.MM1, labels = rownames(PCA.MM1))
```

S.1A

Master Mix 1 Melt Profiles

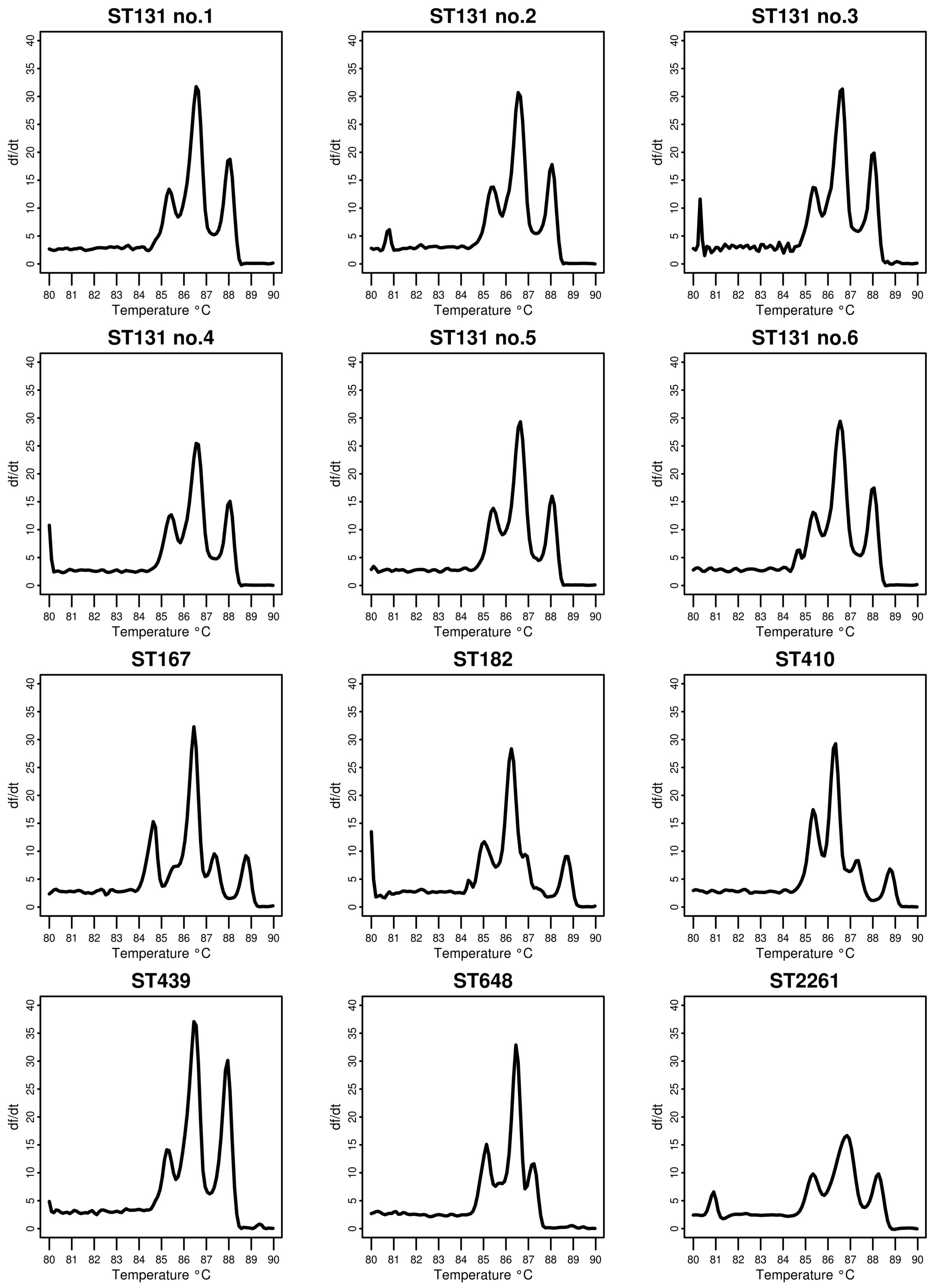

S.1B Master Mix 2 Melt Profiles

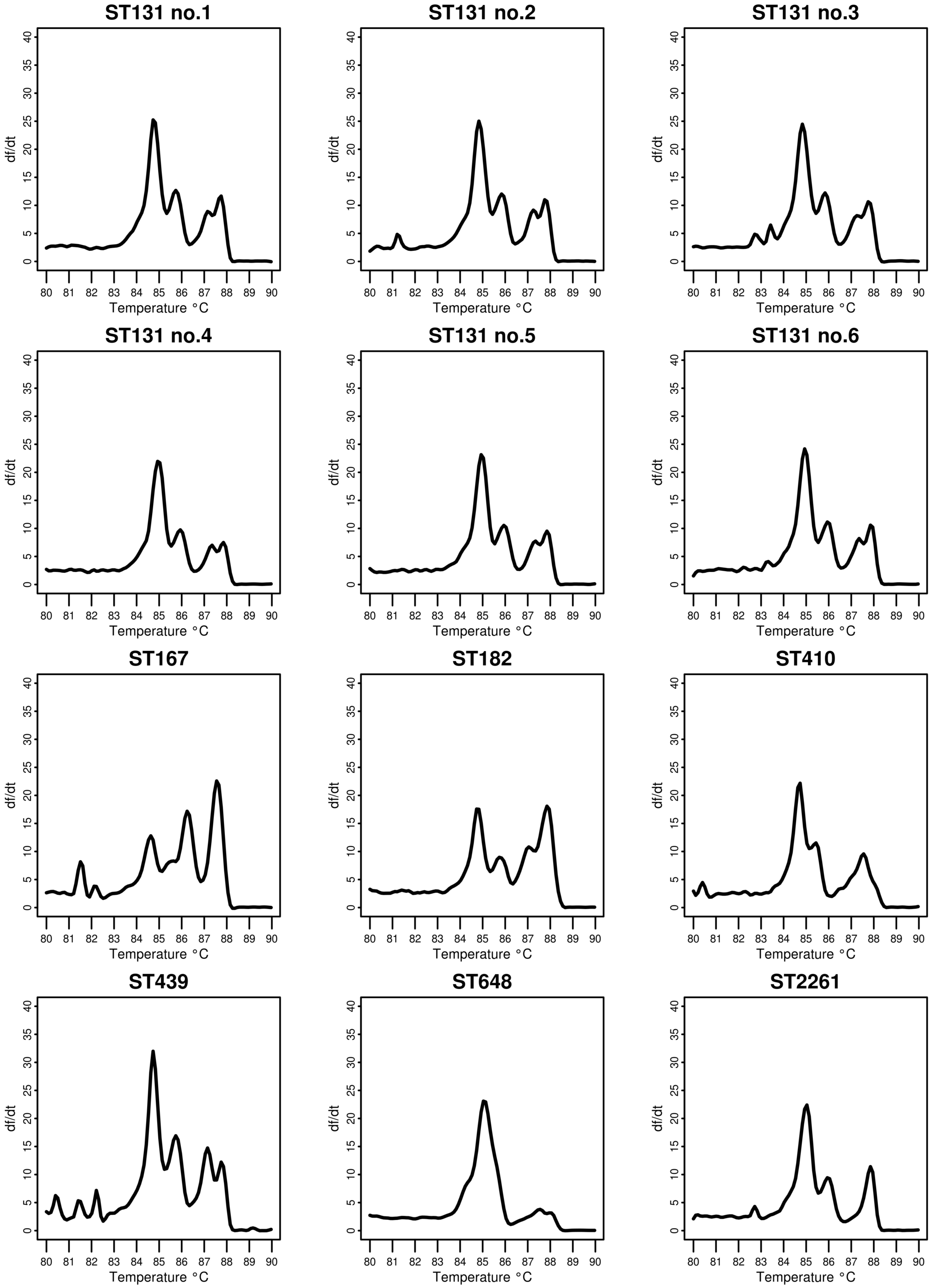

S.1C

ScreenClust Analysis of Master Mix 1

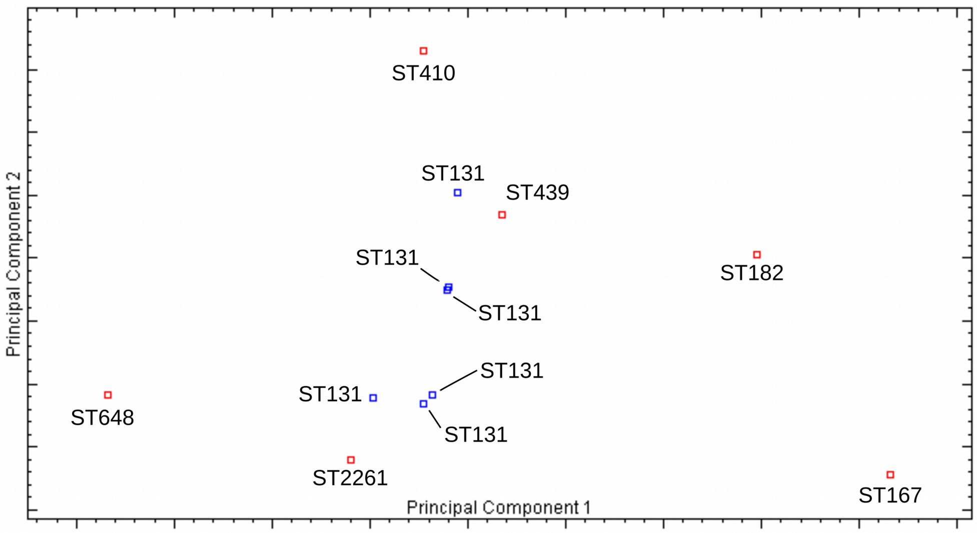

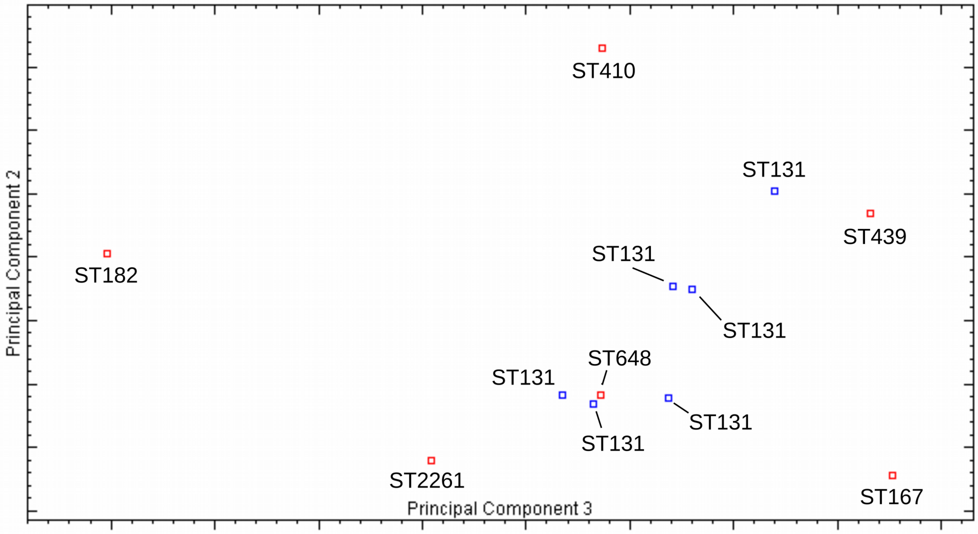

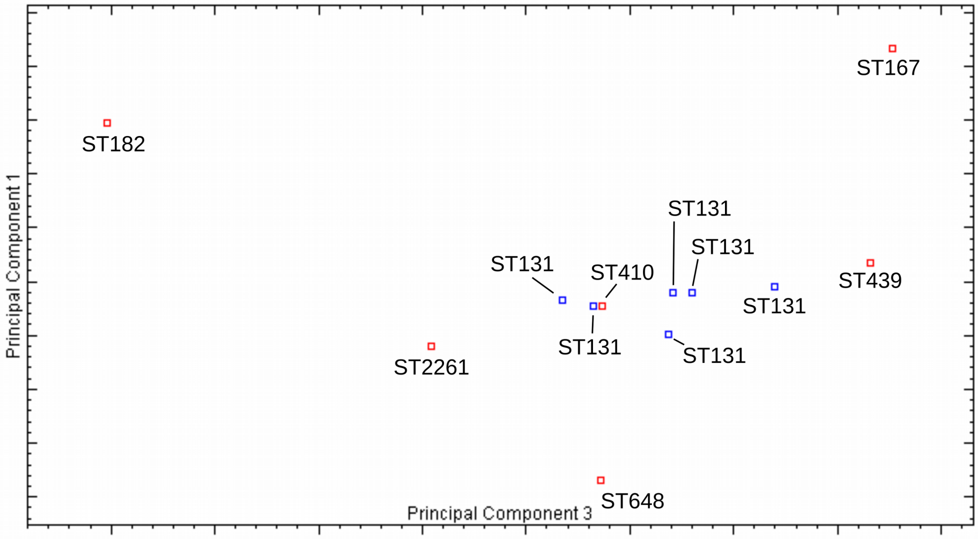

S.1D

ScreenClust Analysis of Master Mix 2

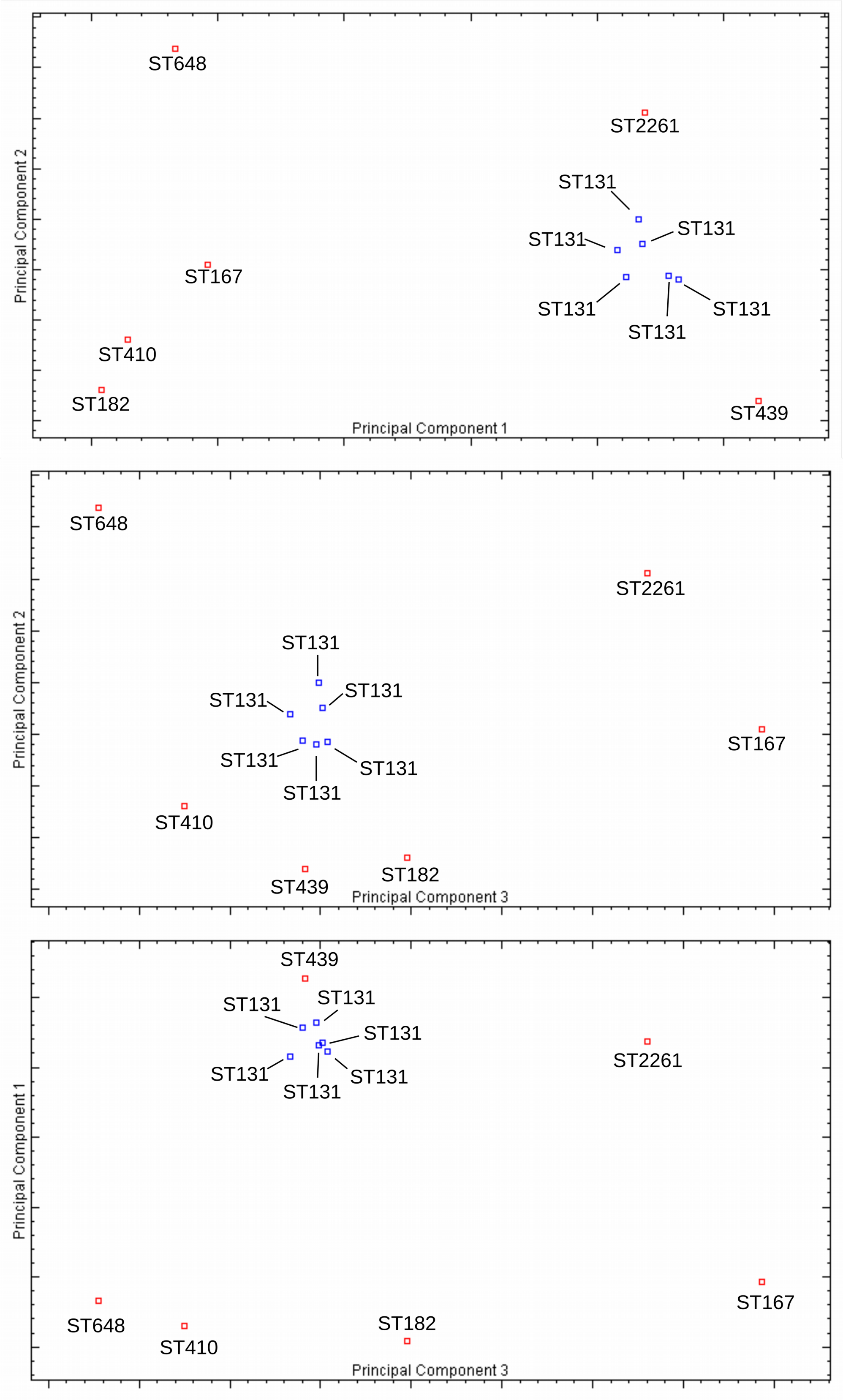

S.Fig 1. Derivative melt curve profiles were compared between 6 ST131 and 6 non-ST131 *E. coli* for the master mixes containing primers for *fumC*, *icd* and *purA* (**A**) and for *adk*, *gyrB*, *mdh* and *recA* (**B**). The same melt profiles were evaluated using the Qiagen ScreenClust^{TM} software (**C** & **D**). Blue boxes indicate ST131 *E. coli* while red boxes indicate non-ST131 *E. coli*. Sequence type labels have been added manually to the PCA plots.

S.2

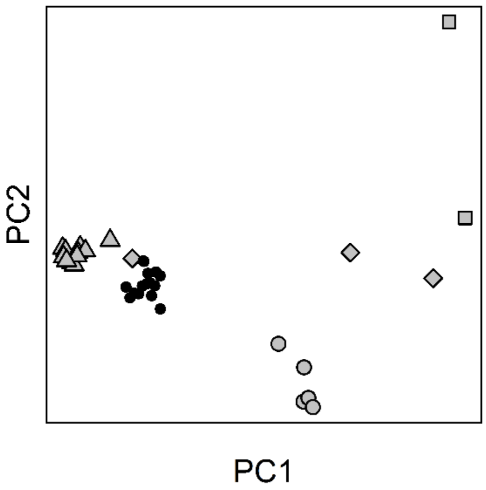

S.Fig 2. A 2D PCA scatterplot using the same data found in figure 2A. This plot represents the different clusters by symbols rather than by color. ST131 *E. coli* are presented as black circles.
